# Mammographic density and markers of socioeconomic status: a cross-sectional study

**DOI:** 10.1186/1471-2407-10-35

**Published:** 2010-02-09

**Authors:** Zoe Aitken, Kate Walker, Bernardine H Stegeman, Petra A Wark, Sue M Moss, Valerie A McCormack, Isabel dos Santos Silva

**Affiliations:** 1 Cancer Research UK Epidemiology and Genetics Group, Department of Epidemiology and Population Health, London School of Hygiene and Tropical Medicine, Keppel Street, London WC1E 7HT, UK; 2 Screening Evaluation Unit, Institute of Cancer Research, 15 Cotswold Rd, Belmont, Sutton, Surrey SM2 5NG, UK; 3 International Agency for Research on Cancer, 150 Cours Albert Thomas, 69008 Lyon, France; 4 Department of Thrombosis and Hemostasis, Leiden University Medical Center, Leiden, The Netherlands; 5 Imperial College London, Department of Epidemiology and Public Health, St Mary's Campus, Norfolk Place, London UK

## Abstract

**Background:**

Socioeconomic status (SES) is known to be positively associated with breast cancer risk but its relationship with mammographic density, a marker of susceptibility to breast cancer, is unclear. This study aims to investigate whether mammographic density varies by SES and to identify the underlying anthropometric, lifestyle and reproductive factors leading to such variation.

**Methods:**

In a cross-sectional study of mammographic density in 487 pre-menopausal women, SES was assessed from questionnaire data using highest achieved level of formal education, quintiles of Census-derived Townsend scores and urban/rural classification of place of residence. Mammographic density was measured on digitised films using a computer-assisted method. Linear regression models were fitted to assess the association between SES variables and mammographic density, adjusting for correlated variables.

**Results:**

In unadjusted models, percent density was positively associated with SES, with an absolute difference in percent density of 6.3% (95% CI 1.6%, 10.5%) between highest and lowest educational categories, and of 6.6% (95% CI -0.7%, 12.9%) between highest and lowest Townsend quintiles. These associations were mainly driven by strong negative associations between these SES variables and lucent area and were attenuated upon adjustment for body mass index (BMI). There was little evidence that reproductive factors explained this association. SES was not associated with the amount of dense tissue in the breast before or after BMI adjustment. The effect of education on percent density persisted after adjustment for Townsend score. Mammographic measures did not vary according to urban/rural place of residence.

**Conclusions:**

The observed SES gradients in percent density paralleled known SES gradients in breast cancer risk. Although consistent with the hypothesis that percent density may be a mediator of the SES differentials in breast cancer risk, the SES gradients in percent density were mainly driven by the negative association between SES and BMI. Nevertheless, as density affects the sensitivity of screen-film mammography, the higher percent density found among high SES women would imply that these women have a higher risk of developing cancer but a lower likelihood of having it detected earlier.

## Background

Higher socioeconomic status (SES) has been shown to be associated with increased breast cancer incidence in a large number of studies [1-7] although recently there has been some evidence of a narrowing of the SES differential over time [3]. This gradient in risk, present in both developed and less developed countries [1,3,6], has been found with individual-level measures of SES such as income, occupation, social class and level of education, as well as area of residence-level measures incorporating several social and material deprivation indicators. These SES variables are likely to be proxies for aetiologically relevant factors such as anthropometric, reproductive and lifestyle variables [4] and, more recently, uptake of mammographic screening [8,9], but there is conflicting evidence whether the observed SES gradient in breast cancer is independent of known breast cancer risk factors [2,4,5].

Breast density corresponds to the amount of fibro-epithelial tissue in the breast which is shown as radiographically dense tissue on a mammogram, as opposed to the fatty tissue which appears as radiographically lucent (non-dense) tissue. Breast density, expressed either as dense area or percent density, is a strong marker of susceptibility to breast cancer [10] although the biological mechanisms underlying this association are unclear. Studies investigating density correlates often adjust for measures of SES, but few [11-13] have assessed whether there is a gradient in mammographic density with SES, and only one [11] was able to examine the associations before and after adjustments for other density correlates, using a continuous measurement of percent density. None of these three previous studies [11-13] examined the separate effects of SES on mammographic dense and lucent tissues.

The finding that mammographic density has a similar gradient with SES as breast cancer would provide further evidence that density is a marker of exposure to breast cancer risk factors. Furthermore, as mammographic density decreases the sensitivity of screen-film mammography [14,15], knowledge of the existence of a SES gradient in density may help to identify groups of women who may require different screening strategies for early detection of breast cancer.

The aims of this study were to assess how mammographic density varies by various individual and area-level measures of SES and to identify the underlying anthropometric, lifestyle and reproductive factors leading to this variation.

## Methods

### Study Participants

The Mammography, Oestrogens and Growth factors (MOG) study is an observational study of mammographic density conducted within the intervention arm of the Age trial of mammographic screening in young women, in which about 54,000 women were randomised to receive yearly mammographic screening from ages 40 to 48 [16]. Cancer-free women who were enrolled in the Age trial and were regularly attending screening were invited to participate between 2000 and 2003 and were sent a baseline questionnaire on demographic, anthropometric, lifestyle and reproductive characteristics, and asked to provide a blood sample. Over 8,000 women from 17 screening centres in England and Wales were enrolled into the MOG study.

A sub-sample of 529 (out of 800 eligible) pre-menopausal Caucasian MOG women, who were not on oral contraception or hormone replacement therapy (HRT) at the time of the original baseline questionnaire, participated in a subsequent study on endogenous hormones and mammographic density. All participants confirmed that they were still not on oral contraception or HRT and provided repeat urine samples throughout their menstrual cycles. They were also asked to complete a follow-up postal questionnaire a few years later (in May 2008) with additional questions on their reproductive history and highest achieved level of formal education. Mammograms for all screening rounds were successfully retrieved from relevant screening centres in Britain for 499 of the 529 women, and films closest to the time of collection of the urine sample were read as part of a previous study [17]. The 487 women who had provided complete reproductive, anthropometric and demographic data in the baseline questionnaire were included in the present analysis. Of these, 369 (76%) also returned a completed follow-up questionnaire. The study was approved by all relevant ethics committees and all participants provided written informed consent.

### Measures of SES

Educational level was used as a measure of SES at the individual level. The follow-up questionnaire collected information on each woman's highest achieved level of formal education, which was analysed using 3 categories: none to GCSE (equivalent to 12 years or less of formal education), further (A-level or vocational training) and higher (university education).

Each woman's socioeconomic circumstances were also ascertained using the Townsend deprivation index, an area-level measure based on either Lower-level Super Output Areas (LSOA) or wards. England and Wales consist of 34,378 LSOA units comprising a target population of 1,500 each, but of just 8,850 wards. Our main analysis used SES indicators based on LSOA geographical units, which were linked to each woman using the postcode of her usual residence at the time of mammography. There were very few women in our study within the same geographical unit, with 89% of LSOA units containing a single woman. The Townsend index of deprivation is based on the combination of the standardised scores (z-scores) of four 2001 Census variables: unemployment, household overcrowding, non-home ownership and non-car ownership [18]. Townsend index was categorised into fifths of its England and Wales general population distribution, ranging from 1 ('deprived') to 5 ('affluent'). To check the sensitivity of our findings to choice of deprivation index and geographical unit, analyses were repeated using ward level fifths of Townsend and Carstairs indices of deprivation. Carstairs index is a composite measure similar to Townsend index but based on the following 2001 Census-derived variables: male unemployment, household overcrowding, non-car ownership, and percentage in social class IV or V (manual and unskilled workers) [19]. In addition to the composite scores of deprivation, a woman's area of residence was also classified according to a Census-derived urban/rural indicator, of whether the nearest settlement had a population greater than 10,000 people (urban) or less than 10,000 people (rural) [20].

### Mammographic Density Measurements

Mammograms were digitised using an Array 2905 laser digitiser (optical density range 0 to 4.0, 12-bit depth, pixel size 75 μm) (Array Corporation Europe, Roden, the Netherlands). Medio-lateral oblique (MLO) views were preferentially selected as they were taken in all screening rounds (MLO views were missing only for four women (1%) and their cranio-caudal (CC) views were used instead). Mammographic density measurements were performed using the Cumulus software, which is based on the interactive threshold method [21]. Breast area was defined by delimiting the skin edge and masking the pectoral muscle and any other non-breast tissue areas. The total breast area was then classified into dense and lucent tissues using an interactive greyscale threshold, from which percent density (100 × dense area/total breast area) and absolute measures of dense and lucent areas were calculated. For each woman the measurements were performed independently for her left and right breasts. Films were randomly ordered and masked to remove all personal identifiers and ensure blinding of the reader to participant and image details. A random sample of 10% of images was re-read independently, with high reliability between readings (intra-class correlation coefficient: 0.92 (95% confidence interval (CI): 0.90, 0.94)).

### Statistical Analysis

To minimise measurement error, the means of each woman's left and right readings for percent density, absolute dense and lucent areas were used. Square root transformations of density measures were performed to ensure normality of residuals. Linear regression models were fitted to generate estimates of mean differences in transformed density values between categories of each SES variable. These are presented in the figure as multiples of the standard deviation (SD). P-values (P_t_) were calculated to assess whether there were linear trends in the mean transformed density values across ranked categories of educational level and fifths of socioeconomic deprivation. To facilitate interpretation of results mean differences were back-transformed from the square root transformed estimates to the original density scales, with reference values corresponding approximately to median values in the study population (i.e. percent density 30%, dense area 35 cm^2^, lucent area 90 cm^2^). Associations between individual- and area-level measures of SES were assessed, and their mutually-adjusted effects on density measures were estimated.

The associations of all correlates with SES and mammographic density variables were examined by performing linear regressions, where the outcomes were continuous, or Chi-squared (X^2^) tests of categorical outcomes. Density measures were square root transformed, body mass index (BMI) was log-transformed and quintiles of Townsend score were used. Models were adjusted for age at mammography (and, if appropriate, mammographic view). To identify the underlying correlates leading to variations in mammographic density by SES, linear regression models were fitted adding variables sequentially. Models were first fitted adjusting only for age at mammography and mammographic view (minimally-adjusted models), then adjusting additionally for BMI (BMI-adjusted models) and, finally, adjusting further for height, smoking status, number of full-term pregnancies and age at first full-term pregnancy (fully-adjusted models). These variables were treated as categorical (categories as defined in Table 1). Further adjustment for family history of breast cancer and lifetime breast feeding had no effect on the point estimates, therefore these variables were not included in the regression models presented in the tables and figures. The analyses were conducted in Stata, version 10 (College Station, Texas).

**Table 1:** Baseline characteristics of the participants and their association with mammographic percent density

*Participant baseline characteristics*	n	Mean	SD	
Age at mammography (yrs)	487	48.6	1.6	
Weight (kg)	487	67.6	12.9	
Age at menarche (yrs)	194	13.1	1.5	
		**n**	**%**	**Median PD (25^th^, 75^th^)^1^**
	
Smoking	487			
Never		273	56	26.7 (13.7, 43.5)
Ex smoker		152	31	29.5 (14.1, 46.7)
Current		62	13	27.2 (10.5, 42.7)
Height (m)	487			
<1.60		158	32	24.9 (13.3, 43.8)
1.60-1.64		137	28	26.9 (12.3, 43.5)
1.65-1.69		110	23	28.6 (15.4, 43.4)
≥ 1.7		82	17	31.1 (18.2, 43.0)
BMI (kg/m^2^)	487			
<22		115	24	46.0 (32.1, 61.6)
22-24.9		145	30	31.5 (19.5, 43.1)
25-27.9		115	24	22.1 (11.6, 37.4)
≥ 28		112	23	9.5 (4.0, 21.6)
Number of full-term pregnancies	487			
0		63	13	32.9 (10.4, 53.6)
1-2		297	61	28.7 (14.2, 45.6)
3+		127	26	23.5 (13.8, 39.0)
Age at first full-term pregnancy^2^	424			
<24 years		140	33	22.7 (12.0, 37.8)
24-28.9 years		160	38	26.3 (14.6, 42.4)
≥ 29 years		124	29	31.6 (16.1, 49.7)
Family history of breast cancer	487			
Yes		53	11	28.6 (15.8, 46.5)
No		434	89	27.5 (13.7, 43.4)
Lifetime breast feeding^2,3^	326			
Never		55	17	21.6 (9.4, 37.9)
1-6 months		90	28	28.4 (13.3, 39.7)
7-12 months		60	18	30.0 (15.5, 52.6)
≥ 13 months		121	37	32.1 (16.4, 47.2)

^1 ^Median percent mammographic density (%) with 25^th ^and 75^th ^percentiles

^2 ^Among parous women only

^3 ^Available only for the 369 women who completed the follow-up questionnaire, 326 of whom were parous

The analyses for the area-level SES measures included all women with mammograms and complete baseline questionnaires. However, analyses of educational level were only conducted in the subset (76%) who returned a completed follow-up questionnaire. Women with missing values for educational level were compared to those with such information in terms of their area-level socioeconomic variables, mammographic readings and distribution of anthropometric, reproductive and lifestyle variables. In addition to a complete case analysis, missing values for educational level were imputed using a woman's available data on Townsend scores, urban indicator, age at mammography, height, weight, number of full-term pregnancies, age at first full-term pregnancy, smoking status and mammographic density by applying the Multiple Imputation by Chained Equations (MICE) method, sampling from the posterior predictive distribution with five imputed datasets [22].

## Results

### Participant Characteristics

The analysis included 487 women, characteristics of whom are summarised in Table 1. By design all women were still pre-menopausal, and not on oral contraceptives or hormone therapy, at the time of their mammography. About half of the participants were overweight (BMI ≥ 25 kg/m^2^). Most (87%) women were parous and, of these, 33% had their first full-term pregnancy before the age of 24 and 37% breastfed for over one year. A larger proportion of women in our sample (60%) than in the general population (40%) lived in the two most affluent area-level quintiles in England and Wales (Table 2). Only 13% of our sample lived in rural areas. Thirty-eight percent of the study subjects had a University degree, with an equally high proportion educated no higher than to GCSE (Table 2).

**Table 2:** Mammographic and socioeconomic characteristics of the participants and their association with mammographic percent density

*Measures of mammographic density*	n	Median	25^th ^percentile	75^th ^percentile	
Dense area (cm^2^)	487	35.3	18.4	51.7	
Lucent area (cm^2^)	487	92.4	60.5	134.7	
Percent density (%)	487	27.7	13.7	43.5	
***Measures of socioeconomic status***		**n**	**%**		**Median PD (25^th^, 75^th^)^1^**
	
Townsend score^2^	487				
Q5 (affluent)		160	33		31.1 (16.4, 46.5)
Q4		133	27		29.0 (15.2, 46.0)
Q3		99	20		25.2 (9.4, 41.8)
Q2		56	12		23.4 (11.2, 40.1)
Q1 (deprived)		39	8		21.6 (11.7, 34.6)
Highest education level	369				
Higher (University)		139	38		33.0 (17.7, 51.6)
Further (A-level/vocational)		82	22		23.6 (11.9, 40.6)
None to GCSE		148	40		25.2 (11.6, 38.6)
Urban/Rural indicator	487				
Rural (Population <10,000)		61	13		28.1 (15.8, 46.0)
Urban (Population ≥ 10,000)		426	87		27.6 (12.9, 43.1)

^1 ^Median percent mammographic density (%) with 25^th ^and 75^th ^percentiles

^2 ^Q1 to Q5 refer to population fifths of Townsend score at LSOA level

Variables examined for associations with SES and density variables included age, BMI, height, smoking, number of full-term pregnancies, age at first full-term pregnancy, family history of breast cancer and lifetime duration of breast feeding. BMI was negatively associated with percent density, reflecting both a negative association with dense area and a strong positive association with lucent area (P_t _< 0.001 for all; Table 3). BMI was also strongly associated with Townsend quintiles (P_t _= 0.001), with women living in the most deprived areas having the highest BMI, but there was no clear gradient with educational levels or urban/rural indicator, although BMI appeared to be slightly higher in women of lowest educational levels and living in urban areas (Table 4). There were associations between the following reproductive variables and at least one measure of SES: a positive association between age at first full-term pregnancy and both Townsend quintiles and educational level (X^2 ^test P < 0.001 for both); a negative association of number of full-term pregnancies with educational level only (X^2 ^test P = 0.006); and a positive association between lifetime breast feeding and educational level only (X^2 ^test P < 0.001) (results not shown). There were also associations between reproductive factors and density measures: a positive association between age at first full-term pregnancy and percent density (P_t _= 0.003); a negative association between number of full-term pregnancies and percent density (P_t _= 0.03); and a positive association between lifetime breast feeding and percent density (P_t _= 0.001).

**Table 3:** Differences in mammographic density measures across categories of BMI

		Percent density^1,2^	Dense area^3^	Lucent area^4^
BMI (kg/m^2^) (n = 487)	<22	0	0	0
	22-24.9	-10.6 (-14.0,-6.9)	-6.0 (-10.8,-0.8)	36.2 (24.7,48.2)
	25-27.9	-17.3 (-20.1,-14.1)	-10.9 (-15.5,-5.9)	66.4 (52.8,80.6)
	≥ 28	-24.6 (-26.4,-22.5)	-19.4 (-23.1,-15.3)	131.6 (115.3,148.4)
P-value for linear trend		P_t _< 0.001	P_t _< 0.001	P_t _< 0.001

^1 ^Linear regression models adjusted for age at mammography and mammographic view

^2 ^For a reference value of 30%

^3 ^For a reference value of 35 cm^2^

^4 ^For a reference value of 90 cm^2^

**Table 4:** Association of SES indicators with BMI

SES Variable		Ratio^1 ^(95% CI)	P-value^2^
Townsend score	Q5^3 ^(affluent)	1	
(n = 487)	Q4	1.01 (0.97, 1.05)	
	Q3	1.04 (0.99, 1.08)	P_t _= 0.001
	Q2	1.07 (1.01, 1.12)	
	Q1 (deprived)	1.08 (1.02, 1.15)	
Highest educational level	Higher	1	
(n = 369)	Further	1.03 (0.98, 1.08)	P_t _= 0.15
	None/GCSE	1.03 (0.99, 1.07)	
Urban/Rural indicator	Rural (≥10,000)	1	P_t _= 0.26
(n = 487)	Urban (<10,000)	1.03 (0.98, 1.08)	

^1 ^Ratios (and 95% confidence intervals) of geometric mean BMI for each category of SES measure relative to baseline (highest SES category), adjusted for age at mammography

^2 ^P-value for linear trend in BMI across categories of a given SES variable

^3 ^Q1 to Q5 refer to the England and Wales general population fifths of Townsend score at LSOA level

### Main Findings

**Figure 1:**
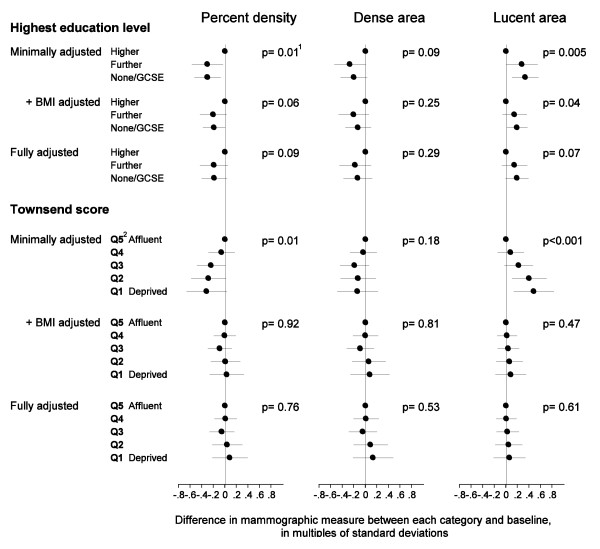
**Differences in mammographic density measures across categories of various SES variables**.^1 ^P-value for linear trend in mammographic values across categories of a given SES variable^2 ^Q1 to Q5 refer to the England and Wales general population fifths of Townsend score at LSOA level

Minimally-adjusted analyses revealed a positive association between percent density and educational level (P_t _= 0.01), with women in the highest educational category having a higher absolute percent density by 6.3% (95% CI 1.6%, 10.5%) compared to the lowest category (Figure 1; Table 5). This positive association between educational level and percent density reflected a negative correlation with lucent area, and to a lesser extent a positive correlation with dense area. Although attenuated on adjustment for BMI, a weak association between level of education and percent density persisted independently of BMI, with an absolute difference in percent density of 4.0% (95% CI-0.1%, 7.9%, P_t _= 0.06). Further adjustment for other covariates in the fully-adjusted model had no effect on the education-percent density gradient (4.0%, 95% CI -0.7%, 8.3%, P_t _= 0.09).

**Table 5:** Differences in mammographic density measures across categories of various SES variables

SES Variable	Percent density^1^	P-value^2^	Dense area^3^	P-value^2^	Lucent area^4^	P-value^2^
***Highest education level***						
Minimally-adjusted^5^						
Higher	0		0		0	
Further	-6.3 (-11.3,-0.8)	0.01	-6.2 (-11.8,0.0)	0.09	15.8 (0.0,32.8)	0.005
None/GCSE	-6.3 (-10.5,-1.6)		-4.7 (-9.6,0.7)		19.3 (5.6,33.8)	
+ BMI-adjusted^6^						
Higher	0		0		0	
Further	-4.3 (-8.8,0.6)	0.06	-4.8 (-10.3,1.3)	0.25	8.2 (-3.7,20.8)	0.04
None/GCSE	-4.0 (-7.9,0.1)		-3.1 (-8.0,2.2)		10.7 (0.6,21.5)	
Fully-adjusted^7^						
Higher	0		0		0	
Further	-4.1 (-8.8,1.1)	0.09	-4.2 (-10.1,2.3)	0.29	8.1 (-4.3,21.4)	0.07
None/GCSE	-4.0 (-8.3,0.7)		-3.2 (-8.6,2.8)		10.7 (-0.8,22.9)	
***Townsend score***^8^						
Minimally-adjusted^5^						
Q5 (affluent)	0		0		0	
Q4	-1.4 (-6.0,3.8)		-0.9 (-6.2,4.7)		4.3 (-8.0,17.5)	
Q3	-5.1 (-9.8,0.2)	0.01	-4.4 (-9.8,1.4)	0.18	12.2 (-1.7,27.1)	<0.001
Q2	-6.0 (-11.5,0.3)		-3.0 (-9.6,4.3)		23.1 (5.4,42.2)	
Q1 (deprived)	-6.6 (-12.9,0.7)		-3.3 (-10.8,5.2)		28.0 (7.3,50.7)	
+ BMI-adjusted^6^						
Q5 (affluent)	0		0		0	
Q4	-0.2 (-4.2,4.2)		-0.1 (-5.1,5.3)		0.9 (-8.4,10.6)	
Q3	-2.0 (-6.2,2.6)	0.92	-2.2 (-7.4,3.5)	0.81	2.1 (-8.0,12.7)	0.47
Q2	0.1 (-5.2,6.0)		1.3 (-5.4,8.7)		3.2 (-9.1,16.5)	
Q1 (deprived)	0.6 (-5.6,7.5)		1.8 (-6.0,10.5)		4.4 (-10.0,19.9)	
Fully-adjusted^7^						
Q5 (affluent)	0		0		0	
Q4	0.1 (-4.0, 4.6)		0.2 (-4.9,5.6)		0.2 (-9.1,10.1)	
Q3	-1.2 (-5.7, 3.5)	0.76	-1.2 (-6.6,4.8)	0.53	1.3 (-9.1,12.3)	0.61
Q2	0.8 (-4.7, 6.8)		2.1 (-4.8,9.7)		2.4 (-10.2,15.9)	
Q1 (deprived)	1.8 (-4.7, 9.1)		3.1 (-5.1,12.3)		3.2 (-11.6,19.3)	
***Urban/Rural indicator***						
Minimally-adjusted^5^						
Rural (<10,000 people)	0	0.34	0	0.66	0	0.17
Urban (≥ 10,000 people)	-2.8 (-8.0, 3.0)		-1.4 (-7.3,5.1)		10.4 (-4.3,26.2)	
+ BMI-adjusted^6^						
Rural (<10,000 people)	0	0.98	0	0.92	0	0.71
Urban (≥ 10,000 people)	-0.1 (-4.7, 4.9)		0.3 (-5.5,6.6)		2.1 (-8.5,13.4)	
Fully-adjusted^7^						
Rural (<10,000 people)	0	0.81	0	0.69	0	0.81
Urban (≥ 10,000 people)	0.6 (-4.2,5.8)		1.3 (-4.7,7.8)		1.4 (-9.5,13.0)	

^1 ^For a reference value of 30%

^2 ^P-value for linear trend in mammographic values across categories of a given SES variable

^3 ^For a reference value of 35 cm^2^

^4 ^For a reference value of 90 cm^2^

^5 ^Adjusted for age at mammography and mammographic view

^6 ^Additionally adjusted for BMI

^7 ^Further adjusted for height, smoking status, age at first full-term pregnancy and number of full-term pregnancies (see Statistical Methods)

^8 ^Q1 to Q5 refer to the England and Wales general population fifths of Townsend score at LSOA level

The association between percent density and Townsend quintiles, at LSOA level, followed a similar pattern to that observed for education, with percent density being highest for women living in the least deprived areas, with an absolute difference of 6.6% (95% CI -0.7%, 12.9%) between the highest and lowest quintiles of SES (P_t _= 0.01) (Figure 1; Table 5). The positive association between area-level SES and percent density was driven almost entirely by the negative association between area-level SES and lucent area (P_t _< 0.001), and was completely attenuated on adjustment for BMI, the absolute difference being reduced to -0.6% (95% CI -7.5%, 5.6%, P_t _= 0.92). Further adjustment for other correlates of density had little effect on the area-level SES-density gradient.

There was no evidence of an association between urban/rural indicator and percent density or dense area in any of the models, but there was a non-significant greater lucent area by 10.4 cm^2 ^(95% CI -4.3 cm^2^, 26.2 cm^2^, P_t _= 0.17) for women living in urban areas relative to those living in rural areas in the minimally-adjusted model, and again this increase was attenuated on adjustment for BMI (Table 5).

Educational level was positively associated with area-level SES (P_t _= 0.02). Inclusion of both of these SES variables in the same regression model, together with BMI, showed that the effect of educational level persisted, with an absolute difference in percent density of 4.0% (95% CI -0.2%, 7.9%, P_t _= 0.06) between highest and lowest educational levels, while the effect of Townsend score was attenuated to a difference in percent density of -0.5% (95% CI -10.0%, 7.7%, P_t _= 0.51) between highest and lowest quintiles of SES (Table 6).

**Table 6:** Mutually-adjusted differences in mammographic density measures across education and Townsend categories

SES Variable	Percent density^1^	P-value^2^	Dense area^3^	P-value^2^	Lucent area^4^	P-value^2^
***Minimally-adjusted***^5^						
Level of education						
Higher	0		0		0	
Further	-6.3 (-11.3,-0.7)	0.02	-6.3 (-11.9,-0.0)	0.11	15.0 (-0.7,32.0)	0.01
None/GCSE	-5.9 (-10.2,-1.1)		-4.4 (-9.4,1.0)		17.2 (3.5,31.8)	
Townsend Score^6^						
Q5 (affluent)	0		0		0	
Q4	-2.0 (-7.2,3.8)		-1.7 (-7.5,4.7)		6.4 (-7.9,21.9)	
Q3	-2.4 (-8.0,3.9)	0.38	-1.4 (-7.8,5.6)	0.96	7.7 (-8.0,24.8)	0.04
Q2	-0.7 (-7.9,7.5)		3.3 (-5.1,12.8)		14.7 (-5.6,37.1)	
Q1 (deprived)	-5.1 (-13.5,5.1)		-2.8 (-12.6,8.8)		24.4 (-2.8,55.3)	
***BMI-adjusted***^7^						
Highest education level						
Higher	0		0		0	
Further	-4.4 (-8.9,0.6)	0.06	-4.9 (-10.5,1.2)	0.25	7.9 (-4.0,20.6)	0.05
None/GCSE	-4.0 (-7.9,0.2)		-3.2 (-8.1,2.2)		10.4 (0.1,21.3)	
Townsend score^6^						
Q5 (affluent)	0		0		0	
Q4	-0.9 (-5.5,4.0)		-0.9 (-6.6,5.2)		2.9 (-8.1,14.6)	
Q3	0.2 (-4.9,5.8)	0.51	0.4 (-5.9,7.3)	0.34	-1.0 (-12.7,11.6)	0.73
Q2	3.2 (-3.5,10.5)		5.8 (-2.6,15.2)		2.9 (-12.0,19.2)	
Q1 (deprived)	0.5 (-7.7,10.0)		1.1 (-9.2,12.9)		5.0 (-14.7,26.9)	

^1 ^For a reference value of 30%

^2 ^P-value for linear trend in mammographic values across categories of a given SES variable

^3 ^For a reference value of 35 cm^2^

^4 ^For a reference value of 90 cm^2^

^5 ^Adjusted for age at mammography and mammographic view

^6 ^Q1 to Q5 refer to England and Wales general population fifths of Townsend scores at LSOA level

^7 ^Additionally adjusted for BMI

### Missing Data and Sensitivity Analysis

Analyses at a ward level geography, rather than at the smaller LSOA level, showed weaker associations between area-level SES and density for both Townsend and Carstairs indices, but all associations reflected the same direction and pattern as those observed with the LSOA Townsend scores. There was evidence of an increasing gradient in percent density with increasing area-level SES (Townsend: P_t _= 0.06; Carstairs: P_t _= 0.03), mainly driven by the negative association of SES with lucent area (Townsend: P_t _= 0.001; Carstairs: P_t _= <0.001), which disappeared on adjustment for BMI (results not shown).

Data on educational level were available only for those women who responded to the follow-up questionnaire (76% of the sample population). Respondents differed from non-respondents in terms of the SES level of their area of residence, with non-respondents living in more deprived areas than respondents (P < 0.001). This was also reflected in some of the reproductive factors that are known to be associated with SES, with lower levels of breastfeeding (46% vs. 73%, P < 0.001) and an earlier age at first birth (24.1 vs. 26.3 years, P < 0.001) among the non-respondents. However, linear regression models using imputed educational level generated almost identical point estimates, with slightly narrower 95% confidence intervals, to those presented here for the complete case analysis (data not shown).

## Discussion

The pattern of association of SES with mammographic density observed in this study paralleled known SES gradients in breast cancer risk [1,3,4,6]. Percent density was greatest among women with the highest educational level and those living in the most affluent areas. The difference in percent density between the lowest and highest SES categories (6-7%, 4% adjusted for BMI) was comparable to, for example, the influence of HRT on density of 4-5% [11,23]. This SES density differential translates to approximately a 8-12% difference in breast cancer risk [10], which is smaller than estimates of SES associated breast cancer differences of 15-36% [4-6]. The effect of level of education on the density measures was mainly apparent in the highest education category. We examined whether women living in affluent areas had higher density levels after controlling for their educational level. The findings showed that the area-level Townsend gradient disappeared after adjustment for educational level while the educational level persisted suggesting that area-level measures are not associated with density beyond the woman's own SES. This contrasts with similar analyses for breast cancer showing that living in affluent areas confers increased risk of breast cancer independently of a woman's own SES [5,7], but it of a is conceivable that these findings may reflect residual confounding due to errors in the measurement of a woman's own SES. Alternatively, the findings may reflect differences in access to screening between areas of different SES.

The positive association between SES and percent density primarily reflected lower mean BMI in women of higher SES. In our data, there was little evidence of reproductive and lifestyle factors accounting for the SES gradient in percent density, perhaps due to difficulties in fully capturing all relevant reproductive and lifestyle factors, or because BMI and the reproductive factors were correlated. The effect of educational level on percent density, although attenuated, persists after adjustment for BMI and other lifestyle and reproductive factors, which further suggests that there may be unmeasured underlying correlates mediating the SES-density gradient. For instance, data on certain known breast cancer risk factors were not available, in particular alcohol intake [11,24], which could explain some of the SES gradient. BMI was calculated from self-reported height and weight, which is prone to measurement error, and also may not be the best measure of adiposity.

SES was not associated with the amount of dense tissue in a mammogram, which represents the amount of stromal and epithelial tissue in the breast. These findings suggest that although SES differentials in breast cancer risk may be mediated by the processes that give rise to differences in percent density, they are unlikely to be mediated by those solely affecting dense area. Both absolute and relative measures of density have been shown to be associated with breast cancer risk but it is unclear which one is the best predictor of risk [25-27]. Relative measures, such as percent density, do not convey any information about the absolute amount of target cells which are potentially at risk of suffering a malignant transformation [28]. They may, however, predict risk better than absolute measures if the lucent area on a mammogram, which represents the amount of fatty tissue in the breast, also contains information about risk perhaps as a proxy measure of the degree of lobular involution that has occurred [29]. BMI is a major determinant of the amount of fatty tissue in the breast and both density and BMI have been found to be independent risk factors for breast cancer [30].

Regardless of the underlying biological mechanisms through which SES may affect density, and hence cancer risk, density affects the sensitivity of screen-film mammography [14,15]. Thus, the higher percent density levels found among high SES women would imply that these women have a higher risk of developing cancer but a lower likelihood of it being detected earlier through conventional screen-film mammography.

Our study benefits from detailed questionnaire data on known density correlates, highly reliable mammographic measurements including separate estimates of absolute amounts of dense and lucent tissues, and data on various area- and individual-level measures of SES. Self-reported educational level is a more reliable measure of long-term SES than self-reported data on other individual-level SES measures (e.g. income or occupational status) as it tends to remain relatively constant throughout a woman's adult life [31]. In contrast, the area-level indicators used in our study were based on a woman's area of residence at the time of her mammography and therefore they may not reflect her lifetime socioeconomic position. Area-level measures of SES have been widely used particularly in the absence of individual measures of SES. People living in the same area tend to experience similar levels of socioeconomic deprivation at a community level [6], particularly if the size of the geographical units is small, even if their individual socioeconomic circumstances may vary. LSOAs have identified larger SES differentials in breast cancer survival than ward geographies [32]. LSOA geographies comprise smaller population units of more consistent size and homogeneous population than ward geographies, and therefore should provide a more valid measure of a woman's SES, being less prone to measurement error. Nevertheless, the analyses at ward level using both the Townsend and Carstairs indices of deprivation demonstrated similar patterns of association between SES and density measures to those shown here for LSOA, thus strengthening the consistency of our findings.

A limitation of the present study is that the SES of the participants was high relative to the general population of England and Wales, which reflects the higher rates of screening uptake among women of higher SES [8,9], but this would not affect the validity of the internal comparisons. The deprivation quintiles used in the analysis were those defined for the whole country and therefore the estimates for the least affluent categories were based on relatively small numbers. Reassuringly, analyses based on study-specific deprivation quintiles provide similar, albeit slightly weaker, gradients in density values with area-level SES measures. However, the analysis of urban and rural areas lacked power to detect associations, as there was very little heterogeneity in our sample (87% of our sample lived in urban areas compared to 79% of the England and Wales general population).

Data on educational level was available only for the 369 (76%) women who completed the follow-up questionnaire. The women with missing data on this variable were of lower area-level SES. Although we cannot rule out the possibility that the results are biased, it is reassuring that the regression using imputed data for education (with Townsend score amongst the variables used to impute educational level) generated very similar point estimates, with slightly narrower 95% confidence intervals, as the complete case analysis.

Our sample comprised pre-menopausal women in their late forties, at a time when density begins to decline markedly. It would be informative to investigate whether similar SES gradients are present at younger ages, when density levels tend to be more stable, and in post-menopausal women, as one previous study found different patterns of association between SES and density according to menopausal status [11]. It would also be informative to examine SES in breast density in populations with different distributions of BMI and reproductive factors. A study of Asian women, mainly post-menopausal, reported a strong positive gradient in density (Tabar patterns IV and V [33]) with educational level [12] whereas Perry et al. found higher density levels (based on BI-RADS categories [34]) in women living in London relative to those living outside [13], but in neither study were the analyses adjusted for reproductive and lifestyle correlates.

## Conclusions

Our study revealed positive associations between SES and percent density, with women in the highest educational level and those living in the most affluent areas having the highest percent density. As a result, these women will not only have a higher risk of developing breast cancer but also a lower probability of having it detected earlier through screen-film mammography. Screen-film mammography is increasingly being replaced by full-field digital mammography, and new volumetric approaches for the measurement of density in digital settings are currently being developed. It would be worthwhile to assess the extent to which the observed SES gradients in density measured with screen-film mammography are also present in digital mammography and how they may impact on breast cancer detection.

## List of Abbreviations Used

BMI: Body Mass Index; CC: Cranio-caudal; CI: confidence interval; LSOA: Lower-level Super Output Area; MLO: Medio-lateral oblique; MOG: Mammography, Oestrogens and Growth Factors (study); SD: standard deviation; SES: socioeconomic status.

## Competing interests

The authors declare they have no competing interests.

## Authors' contributions

Conception and design: IdSS, BHS, ZA, VM; financial support: IdSS, VM; collection of data: ZA, BHS, SMM, IdSS; data analysis: ZA, KW, BHS, PAW; production of first draft of the manuscript: ZA, KW, IdSS; data interpretation and contribution to final version of the manuscript: all authors.

## Pre-publication history

The pre-publication history for this paper can be accessed here:

http://www.biomedcentral.com/1471-2407/10/35/prepub
